# Neurophysiological insights into catecholamine-dependent tDCS modulation of cognitive control

**DOI:** 10.1038/s42003-025-07805-6

**Published:** 2025-03-06

**Authors:** Anna Helin Koyun, Paul Wendiggensen, Veit Roessner, Christian Beste, Ann-Kathrin Stock

**Affiliations:** 1 Cognitive Neurophysiology, Department of Child and Adolescent Psychiatry, Faculty of Medicine, TU Dresden, Dresden, Germany; 2 University Neuropsychology Center, Faculty of Medicine, TU Dresden, Dresden, Germany; 3 German Center for Child and Adolescent Health (DZKJ), partner site Leipzig/Dresden, Dresden, Germany

**Keywords:** Cognitive control, Neuronal physiology, Human behaviour

## Abstract

Goal-directed behavior requires resolving both consciously and subconsciously induced response conflicts. Neuronal gain control, which enhances processing efficacy, is crucial for conflict resolution and can be increased through pharmacological or brain stimulation interventions, though it faces inherent physical limits. This study examined the effects of anodal transcranial direct current stimulation (atDCS) and methylphenidate (MPH) on conflict processing. Healthy adults (*n* = 105) performed a flanker task, with electroencephalography (EEG) used to assess alpha and theta band activity (ABA, TBA). Results showed that combining atDCS with MPH enhanced cognitive control and reduced response conflicts more effectively than atDCS alone, particularly when both conflict types co-occurred. Both atDCS and atDCS + MPH exhibited similar task-induced ABA and TBA modulations in the (pre)supplementary motor area, indicating heightened gain control. Overlapping neuroanatomical effects in mid-superior frontal areas suggest that atDCS and MPH share a common neuronal mechanism of gain control, especially in high-conflict/-demand situations.

## Abstract (web summary)

Overlapping neuroanatomical alpha and theta band effects in mid-superior frontal areas suggest that anodal tDCS and methylphenidate share a common neuronal mechanism of gain control, especially in high-conflict/-demand situations.

## Introduction

Resolving response conflicts is influenced by the brain’s ability to effectively distinguish relevant input from intrinsic noise (including irrelevant stimulus features). This key component of goal-directed behavior and cognitive control particularly relies on the signal-to-noise ratio (SNR), which indicates the processing system’s sensitivity to important inputs versus neural noise^1,2^. Fine-tuning the SNR, namely sharpening the neuronal input/output function (the probability that varying strengths of presynaptic input will trigger a postsynaptic response), describes neuronal gain control mechanisms^1,3^. Enhanced neural gain control is linked to improved response control and overall cognitive, motor, and sensory performance^4–6^. Here, we investigated the effect of modulating gain control onto cognitive control processes, specifically the selection of appropriate actions by prioritizing relevant information over consciously and subliminally perceived cued distractions.

Research indicates that gain control mechanism can be modulated by various pharmacological and non-invasive brain stimulation (NIBS) techniques^1,7–9^, both of which may exhibit ceiling effects as there is a limit to how much gain control can be increased. Combining both approaches in a proof-of-concept study will provide crucial insights into their interactive effects. In the current study, we therefore combined a NIBS method and a psychostimulant, of which both have been shown to modulate the efficacy of gain control mechanisms. Specifically, we used transcranial direct current stimulation (tDCS), which through a low electric current applied to the scalp induces focal changes in cortical excitability (i.e., increase or decrease dependent on electrode polarity), which last about one hour^10,11^. Cortical excitability adjusts neuronal gain control^9,12^, i.e., anodal tDCS (atDCS) induced amplification of spontaneous neuronal firing, and cortical excitability has been associated with modulation of gain control efficacy^12,13^. We applied atDCS to the right inferior frontal gyrus (rIFG) a region critical for response conflict resolution, and action inhibition^14–16^. This region was targeted, as it was shown to reflect response conflict effects specific to the experimental task at hand^6^. Furthermore, the catecholaminergic system underlies effective neural information processing, in that appropriate levels of dopamine (DA) and norepinephrine (NE) are central to conflict processing and resolution^17–19^. Thus, we used methylphenidate (MPH), a psychostimulant that selectively blocks DA/NE reuptake, consequently increasing postsynaptic DA and NE levels^20–22^. Previously, MPH-induced modulations of cognitive control have been attributed to neuronal gain mechanisms and linked to fronto-striatal loops^6,23^. Importantly, the rIFG has been highlighted as a key site for reflecting the modulatory effects of MPH on cognitive control^6^, making it, together with its well-established role in cognitive control, a suitable stimulation target.

In the current literature, there is a notable lack of research combining tDCS with pharmacological interventions in the context of cognitive control. Few studies have explored how these two modulations might interact to influence neural plasticity and reorganization^24–29^, underlining the need for further investigations in this area. While both tDCS and MPH have been studied individually, their combined effects on behavioral accuracy and brain oscillations, particularly during cognitive tasks inducing conscious and subconscious conflicts, have not been investigated. However, both consciously and subconsciously processed information affect controlled behavior^30–33^. To investigate such response conflicts in the current study, we used a combination of a spatial flanker task with preceding subliminal primes^6,34,35^ (full details in the methods section). Gain control facilitates task-relevant processing and modulations via atDCS appear to facilitate the resolution of stimulus-response conflicts^36^. In contrast, MPH-induced increases in gain control were shown to benefit the resolution of subconsciously and consciously induced conflicts differentially^6^.

As a measure of neuronal mechanisms underlying task performance modulations, we focused on both theta and alpha band activity (TBA and ABA). TBA coordinates and integrates neuronal activity in complex networks, reflecting conflict monitoring and response conflicts^37–39^. Whereas, ABA has been implicated in interceding suppression of task-irrelevant information by regulating access to behaviorally relevant information^40,41^. Both ABA and TBA are associated with neuronal gain control mechanisms by facilitating selective noise inhibition and enhancing perceptual and response selection accuracies^42–45^. This dual focus on cognitive and neuronal gain control relevant TBA and ABA will likely provide a comprehensive understanding of the neuronal dynamics underlying gain control modulations via atDCS and MPH.

In this study, we set out to investigate the effects of gain control modulations via atDCS and atDCS + MPH on consciously and subconsciously triggered response conflicts. Presuming that atDCS over the rIFG and MPH administration enhances the SNR in cortical areas relevant to response conflict processing, we hypothesize that (1) consciously and subconsciously induced response conflicts will engage distinct functional neuroanatomical networks (2) behavioral effects will be accompanied by comparable ABA and TBA modulations (reflecting gain control modulations) in mid-superior and inferior-frontal areas^6,29,39^ (3) the combined effects of atDCS and MPH will surpass the individual effects of each technique^29^ (4) combining atDCS with MPH will result in additive effects (rather than doubling) of both stimulation methods^29^, given that the extent to which gain control may be increased underlies a physical limit. The goal was to investigate neuronal gain control as a common neuronal mechanism underlying atDCS and MPH effects. Our neurophysiological analyses were aimed at investigating the neural mechanisms driving behaviorally relevant effects, providing insight into the cortical dynamics that underpin cognitive and behavioral outcomes obtained in this study. Accordingly, functional-neuroanatomical networks underlying consciously and subconsciously induced response conflicts modulated with atDCS and MPH (separately and combined) were examined.

## Results

### Included participants

After initial data inspection, *n* = 12 participants were excluded from all subsequent analyses (behavioral and EEG data). Participants were excluded based on the following criteria: (1) incomplete data due to technical issues (e.g., equipment malfunctions), (2) failure to comply with task instructions (e.g., responding inconsistently or inaccurately beyond acceptable thresholds), and (3) inability to complete study both study appointments (e.g., withdrawal or scheduling conflicts). Additional details are provided in the supplement (section “Additional details on exclusion decisions and the composition of the final sample”).

This resulted in a final sample of *n* = 93 (mean age 25.11 ± 0.315 SEM; 44 females) participants included in all subsequent analyses. Specifically, there were *n* = 45 (mean age 25.20 ± 0.428 SEM; 21 females) participants in the placebo group and *n* = 48 (mean age 25.02 ± 0.465 SEM; 23 females) in the MPH group.

### Behavioral data

#### Task effects

The typical task effects of prime compatibility and flanker congruency (i.e., higher accuracies in congruent/compatible than in incongruent/incompatible trials) were replicated^6,34^. There was an interaction of prime compatibility x flanker congruency (*F*_(1,89)_ = 57.930; *p* < .001; *η*^*2*^_*p*_ = 0.394). Post hoc t-tests revealed significant differences for all possible contrasts (all *p* ≤ .001). In line with previous results, the prime compatibility effect (i.e. compatible – incompatible) was larger in incongruent flankers (10.263% ± 1.167) than in congruent flankers (6.722% ± 0.771) (*t*_(1,75)_ = −7.390; *p* < 001). Additionally, the flanker congruency effect (i.e. congruent – incongruent) was larger in incompatible primes (5.264% ± 0.553) than in compatible primes (1.754% ± 10.234) [*t*_(1,92)_ = −7.346; *p* < 0.001 (one-sided *p*)].

### Pharmacological group and tDCS stimulation effects

The repeated measures ANOVA for accuracy revealed an interaction of tDCS x order of stimulation x prime compatibility x flanker congruency x pharmacological group (*F*_(1,89)_ = 4.132; *p* = .045; *η*^*2*^_*p*_ = 0.044). Next, the interaction effect was separately examined with post-hoc ANOVAs for real vs. sham tDCS appointments.

The post-hoc ANOVA for the active tDCS, but not for the sham tDCS appointment, revealed an interaction of order of stimulation x prime compatibility x flanker congruency x pharmacological group (*F*_(1,89)_ = 9.535; *p* = .003; *η*^*2*^_*p*_ = 0.097). To resolve this interaction, independent samples t-tests were conducted to compare the magnitudes of flanker and prime effects between the pharmacological groups when they received active tDCS. Of note, using those difference effects focusses the post-hoc analyses to the most relevant behavioral performance measures while simultaneously shortening subsequent analyses due to employing contrasts. These comparisons were made separately for participants who received active tDCS during their first appointment and those who received active tDCS during their second appointment. The results show that for participants who received active tDCS on the second appointment, the flanker congruency effect (i.e., congruent – incongruent trials) in case of incompatible primes was larger in the placebo group (7.737% ± 1.734) than in the MPH group (3.728% ± 1.001) [*t*_(43)_ = 1.979; *p* = .027 (one-sided *p*)]. Moreover, the prime compatibility effect (i.e. compatible – incompatible trials) in case of incongruent flankers was also larger in the placebo group (9.165% ± 2.052) than in the MPH group (4.741% ± 1.372) [*t*_(43)_ = 1.775; *p* = 0.041 (one-sided *p*)]. For participants who received active tDCS during their first appointment, no statistically significant differences in the contrasts of interest (flanker and prime effects) were observed between the pharmacological groups [all *p* > 0.051 (one-sided *p*)].

**Fig. 1:**
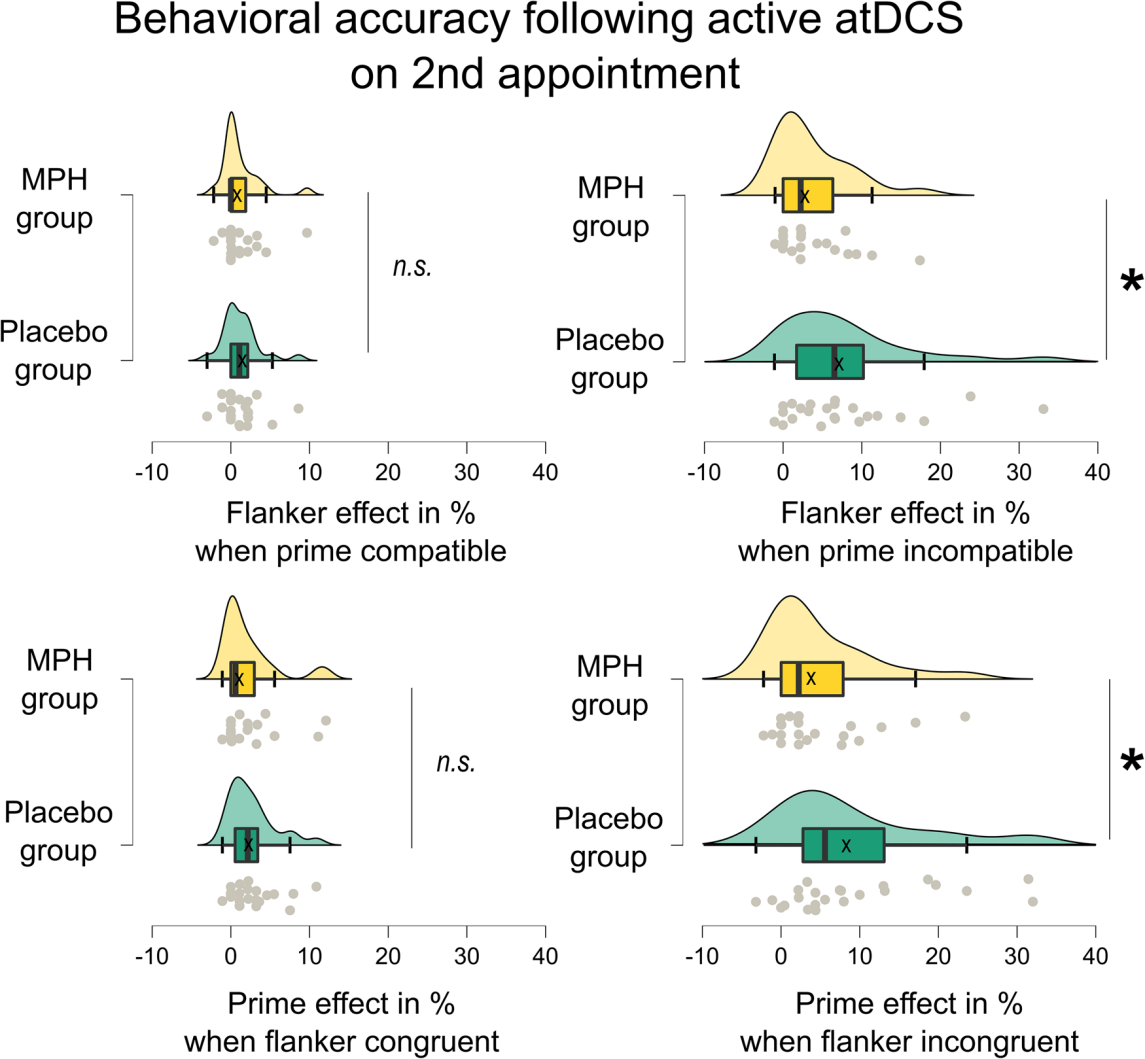
Behavioral accuracy results following active tDCS stimulation on the second appointment. Shown are the prime effects (in percent) for when the flankers were congruent (top row, left plot) and incongruent (top row, right plot), as well as the flanker effect (in percent) for when the primes were congruent (bottom row, left plot) and incongruent (bottom row, right plot). Green boxplots illustrate data for the placebo group and yellow boxplots for the MPH group. The “x” and the horizontal line inside the boxplots indicate the mean and median, respectively. Asterisks indicate significant differences at *p* < 0.05 and error bars represent the 95% confidence intervals. The raincloud plots illustrate the data distribution (above boxplot) and data points (below boxplot) of *n* = 93 participants.

Taken together, significant differences between the pharmacological groups emerged exclusively when active tDCS was applied during the second appointment. This effect was observed only under high response conflict conditions – specifically, in scenarios where the respective other conflict was also present (i.e. when the flanker effect involved an incompatible prime, and the prime effect involved an incongruent flanker). In other words, when active tDCS was applied during the second appointment, the MPH group showed smaller flanker and prime effects compared to the placebo group. Figure 1 illustrates the most relevant behavioral accuracy results. The significance and magnitude of the overall learning/practice effects are reported in the supplementary material (section “Additional details on behavioral data: Learning/practice effects”).

### Response times (RTs)

**Fig. 2:**
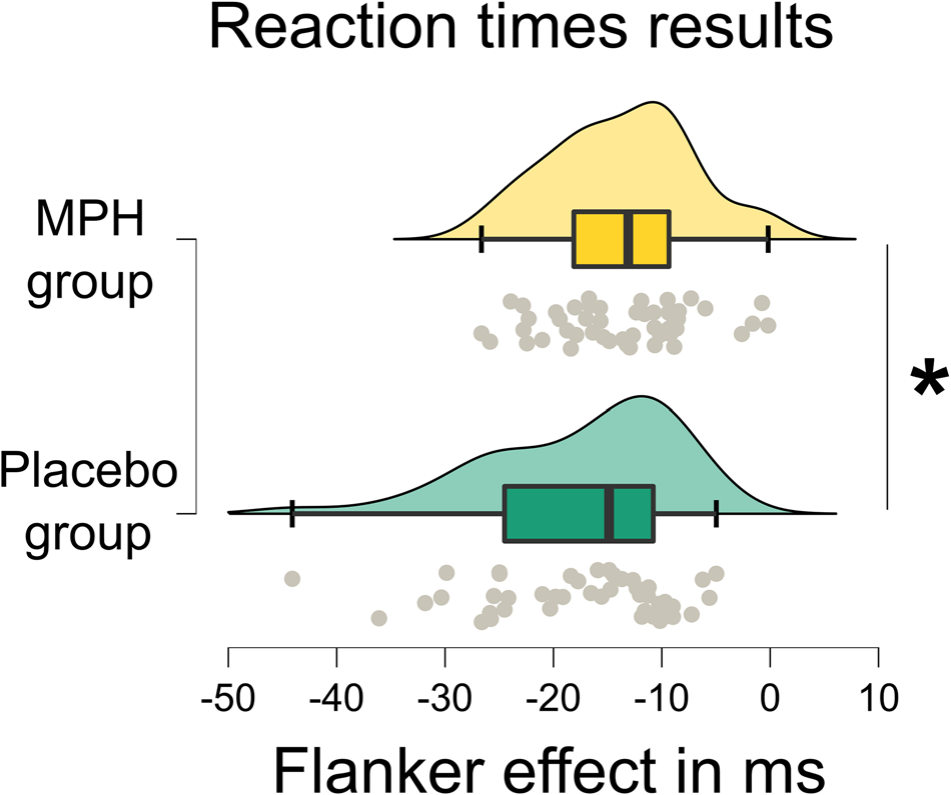
Reaction time results. Depicted is the flanker effect (congruent minus incongruent trials) for both pharmacological groups (MPH and placebo). Data is illustrated in green for the placebo group and in yellow for the MPH group. Asterisks indicate significant differences at *p* < 0.05 and error bars represent the 95% confidence intervals. The “x” and the horizontal line inside the boxplots indicate the mean and median, respectively. The raincloud plots illustrate the data distribution (above boxplot) and data points (below boxplot) of *n* = 93 participants.

The repeated measures ANOVA for RTs showed a main effect of the pharmacological group (*F*_(1,89)_ = 4.914; *p* = 0.029; *η*^*2*^_*p*_ = 0.052), indicating overall faster response times of the MPH group (395.145 ms ± 4.852) as compared to the placebo group (410.583 ms ± 4.995). Importantly, there was an interaction of flanker congruency x pharmacological group (*F*_(1,89)_ = 4.581; *p* = 0.035; *η*^*2*^_*p*_ = 0.049). Post hoc independent samples t-tests show that overall the flanker effect (congruent minus incongruent trials) was larger (more negative) in the placebo group (−17.372 ms ± 1.306) than in the MPH group (−13.709 ms ± 0.933) [*t*_(80.675)_ = −2.282; *p* = 0.013 (one-sided *p*)]. The relevant results are illustrated in Fig. 2.

Further main and interaction effects not involving the pharmacological group factor included the following: There was a main effect of the order of stimulation (*F*_(1,89)_ = 4.557; *p* = .036; *η*^*2*^_*p*_ = 0.049), showing overall faster response times for those who received active tDCS on the second appointment (-395.430 ms ± 4.995) as compared to those on the first appointment (-410.297 ms ± 4.852). Moreover, there was an interaction of tDCS x order of stimulation x flanker congruency (*F*_(1,89)_ = 5.020; *p* = 0.028; *η*^*2*^_*p*_ = 0.053). The interaction was examined with an independent samples t-test comparing the flanker effect after real and sham tDCS between those who received active tDCS on the first vs. those who received active tDCS on the second appointment. The results indicate smaller flanker congruency effects for those who received active stimulation on the second appointment (-9.978 ms ± 0.959) as compared to those who received active tDCS on the first appointment (-12.948 ms ± 0.869) [*t*_(91)_ = -2.2300; *p* = .012 (one-sided *p*)]. There was no difference in the magnitude of the flanker effects on the sham tDCS appointment.

### Neurophysiology

The behavioral differences between both pharmacological groups were only significant when participants received active tDCS stimulation on the second appointment. Based on this, subsequent neurophysiological analyses exclusively investigated differences between the MPH and placebo group when active tDCS was administered on the second appointment.

**Fig. 3:**
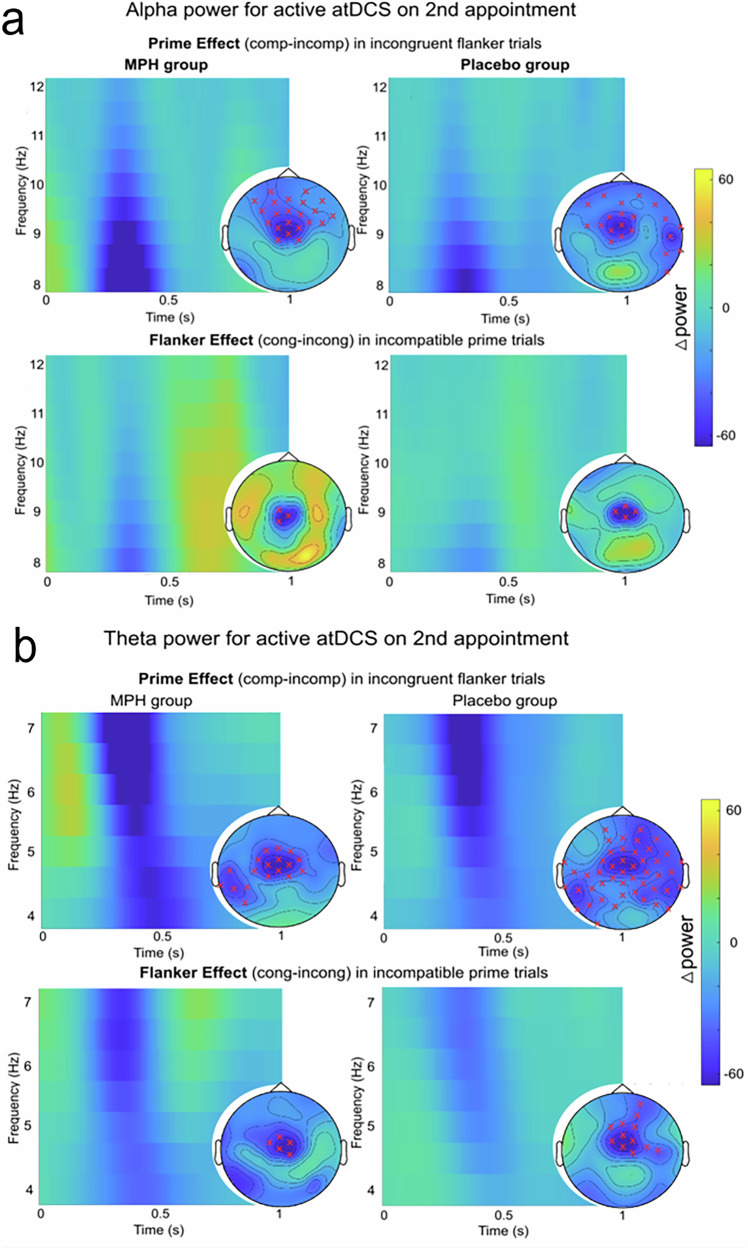
Time-frequency decomposition of *n* = 93 participants. **a** Alpha band time-frequency (TF) analysis and cluster-based permutation testing (CBPT) results. The TF plots display average power differences (0-1000 ms post-target arrow onset) for the prime effect (compatible MINUS incompatible trials) when flankers were incongruent, as well as for the flanker effect (congruent MINUS incongruent trials) when the prime was incompatible. Topographic plots highlight significant electrodes identified in the CBPTs, marked with red “x“s to indicate negative differences. These plots show average power in the significant timeframe (100 to ~600 ms) separately for both MPH and placebo groups, with conflict effects noted in corresponding titles. **b** Theta band time-frequency (TF) analysis and cluster-based permutation testing (CBPT) results. The TF plots display average power differences (0–1000 ms post-target arrow onset) for the prime effect (compatible MINUS incompatible trials) when flankers were incongruent, as well as for the flanker effect (congruent MINUS incongruent trials) when the prime was incompatible. Topographic plots highlight significant electrodes identified in the CBPTs, marked with red “x“s to indicate negative differences. These plots show average power in the significant timeframe (250 to ~750 ms) separately for both MPH and placebo groups, with conflict effects noted in corresponding titles.

In the interval of 0−1000 ms relative to target onset, separate cluster-based permutation tests (CBPTs) were conducted for alpha and theta band activity (ABA and TBA respectively), and MPH and placebo groups. The results revealed significant negative differences in both ABA and TBA between compatible and incompatible primes trials when the flankers were incongruent, as well as between congruent and incongruent flanker trials when the prime was incompatible. Negative power differences indicate that alpha and theta band power were higher in incompatible prime trials compared to compatible ones, and in incongruent flanker trials compared to congruent ones, potentially highlighting greater difficulty, interference, or need for cognitive control effort in these conditions. Further details are provided in the supplement (section “Additional neurophysiological results for participants who received sham tDCS on the first appointment”). The significant electrode locations of the cluster-based permutation tests are visualized in Fig. 3, accompanied by the time-frequency representation (TFRs) of the power differences in the aforementioned contrasts.

**Fig. 4:**
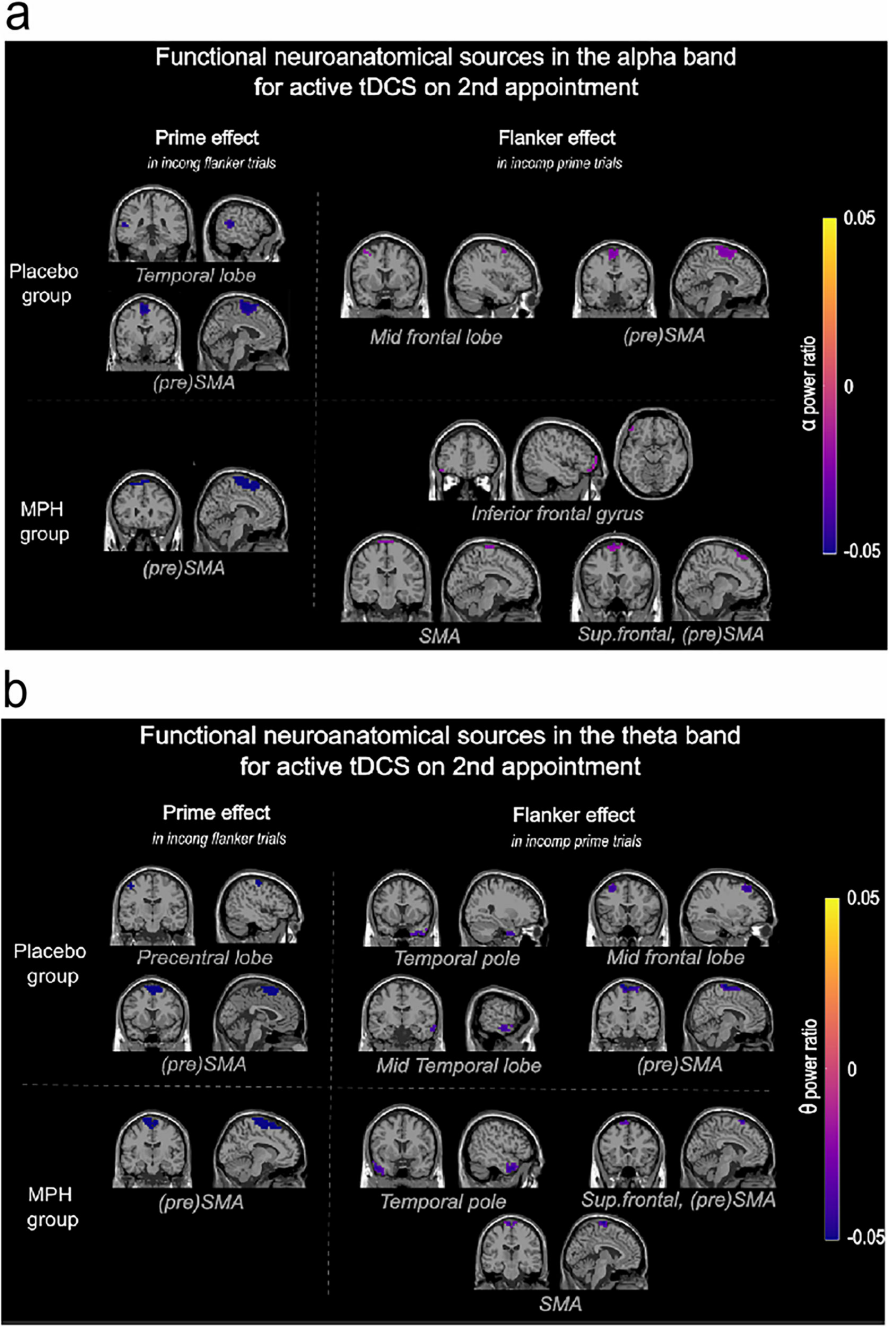
Neuroanatomical clusters of source-localized activity in alpha and theta bands of *n* = 93 participants as identified by the DBSCAN algorithm. The color of the cluster represents the power ratio of the prime effect (when flankers are incongruent) and of the flanker effect (when the prime is incompatible). **a** Clusters identified in the alpha band. **b** Clusters identified in the theta band.

On the source level, the DBSCAN algorithm on the Prime and Flanker effect ratio identified several negative clusters in both alpha and theta bands for both MPH and placebo groups. Negative clusters (i.e., negative power ratios) indicate that ABA and TBA were higher in incompatible prime trials compared to compatible ones, and in incongruent flanker trials compared to congruent ones. The negative ratio reflects the directionality of this difference and suggests that the incompatible prime/incongruent flanker is influencing the processing system more prominently than the compatible prime/congruent flanker under these specific circumstances. An overview of all resulting clusters is provided in Fig. 4.

## Discussion

This study investigated alterations in the functional neuroanatomical sources associated with subconsciously and consciously evoked response conflicts when gain control was experimentally increased. Assuming that atDCS and MPH share a common neuronal mechanism (i.e., gain control), we investigated the modulatory effects of atDCS, MPH, and atDCS + MPH via their effects on interference control and corresponding alpha and theta band oscillations (reflecting gain control). Compared to those who only received tDCS, participants who received both stimulations (i.e., atDCS + MPH) on the second appointment showed lower interference conflict effects, especially when subliminal and conscious conflicts were combined. Using an EEG beamforming approach, we identified the functional neuroanatomical correlates of atDCS-induced, and atDCS + MPH-induced modulations in ABA and TBA, respectively. Across both frequency bands, our results show overlapping neuroanatomical sources when atDCS and atDCS + MPH were applied. Consistent with the assumption that tDCS and MPH share a common underlying neural mechanism [most likely gain control, 4], we show that tDCS separately and combined with MPH modulates TBA in cognitive control-relevant mid-superior-frontal areas. Importantly, we extend these findings by showing similar modulatory effects in the ABA, highlighting the widespread influence of these interventions on cognitive control processes.

The behavioral data indicate clear differences between the pharmacological groups on the active atDCS appointment but not on the sham tDCS appointment. Specifically, when participants received atDCS on their second appointment (i.e., in the presence of learning/practice effects), both the flanker congruency effect and prime compatibility effect were significantly larger in the placebo group compared to the MPH group under high interference conflict conditions (i.e., in the presence of the respective other conflict, i.e., incompatible prime or incongruent flanker trials). While we did not have an a priori hypothesis expecting such learning/practice or order effects, this observation aligns with some of our previous MPH stimulation studies that demonstrated cognitive control-enhancing MPH effects^46,47^, but also hampered behavioral adaptation^48^, and sometimes even a reduction of MPH effects on simple response inhibition and perception-action integration^49–52^ in case of repeated measurements (i.e., after the task had already been performed once without MPH before). Given that the effectiveness of MPH modulation does generally not only depend on the dose, but also on task difficulty and prior learning/practice, it would have been unscientific to have postulated a clear direction of effects a priori. This does however not mean that the obtained effect cannot be interpreted: Given that the participants showed a small, but significant overall improvement in behavioral performance from the first to the second appointment (by 0.9% ± 0.3 and 11 ms ± 25; see supplement for details), it can be assumed that their improvement in accuracy and speed is likely due to learning/practice effects and/or response automatization. Given that we found no significant MPH differences in prime and flanker effects on the first appointment, it is unlikely that MPH produced out data pattern by hampering the initial learning of the task to a relevant degree. Instead, it seems that MPH only made a difference, once a relevant degree of learning / training had taken place (and tDCS stimulation was present, but this will be discussed in further details below). Given that the MPH effects were specific to high-conflict/demand situations, it seems plausible to assume that the combined effects of MPH and tDCS are most pronounced in a situation where easy responses have been extensively trained / automatized, which might potentially make it harder to switch into a cognitive control mode when needed (i.e., when a high-conflict trial pops up). Neurobiologically, the modulatory impact of elevated catecholamine levels on gain control processes may have been further strengthened by increasing cortical excitability via atDCS^13,26,29^, leading to optimal performance. No significant differences were found when atDCS was applied on the first appointment. Our results corroborate previous findings on the effects of MPH [19–22 etc.] and underscore the significant impact of the intricate interplay between atDCS, catecholaminergic stimulation, and the order of tDCS administration onto behavioral outcomes, particularly under challenging conditions (i.e. when the mental effort needed to perform well is high). In the following, we will separately discuss the functional neuroanatomical sources reflecting the prime and flanker effects when gain was modulated (via atDCS) on the second appointment.

### Prime effect

In both frequency bands and pharmacological groups, the prime effect (in incongruent flanker trials) was associated with negative clusters in the (pre)SMA. Agreeing with previous results, TBA in the SMA is elevated when information conflicts and the need for cognitive control is high^29,37,53^. Moreover, TBA in mid-superior frontal regions is essential for cognitive control processes during response control, selection, and execution^29,37,54,55^. This is likely because TBA in these regions is particularly well-suited for integrating sensory information with motor commands^37^.

Notably, ABA is crucial for TBA-associated cognitive control processes and information-gating^56^, and their interplay is central for goal-directed action control. Increased ABA in the SMA likely reflects active engagement in attentional modulation/weighting and cognitive control^56–58^. The combined atDCS and MPH-induced amplification of the signal-to-noise ratio was sufficient to better suppress the processing of distracting information^59^, leading to improved/optimal interference and cognitive control. Mid-superior frontal ABA thus likely reflects the suppression of task-irrelevant information^40,41^. Our results show that after modulation of gain control via atDCS, but not when atDCS and MPH stimulation were combined, ABA additionally increases in an occipito-temporal cluster when the response conflict is high. The occipito-temporal areas have been linked to guiding (visual) attentional selection processes^60,61^. This pattern suggests that participants in the placebo group who experienced higher behavioral interference demonstrated enhanced visual processing of the prime stimulus and subsequently needed higher suppression of distracting information within occipito-temporal regions implemented by ABA. This likely reflects a gain-induced compensatory mechanism to manage increased cognitive load and mental effort to perform well. Our current results support the role of ABA in the temporal lobe as a critical element in moderating interference through selective (visual) attention and filtering out irrelevant or distracting stimuli^40,59^, especially when the cognitive load is high.

### Flanker effect

Similar to the prime effect, the flanker effect (in incompatible prime trials) was associated with negative clusters spanning mid-superior-frontal areas^62^, across both frequency bands and pharmacological groups. When atDCS and MPH were combined, an additional cluster of ABA was detected in the left inferior frontal gyrus (left IFG). This aligns with a recent study^29^, suggesting that the additive effect of both modulations on neuronal gain control likely pushed the mid-superior frontal regions beyond their optimal functioning level, leading to the compensatory recruitment of higher processing capacities (i.e., the left IFG). Activity in the left IFG highlights its essential function in detecting and resolving conflicts at both the response and motor response levels^63,64^ and response control^65^.

In contrast to the prime effect, the flanker effect and associated interference conflict appear to engage a more distributed functional neuroanatomical network, particularly in the theta band. When modulating gain control with atDCS the flanker effect was additionally associated with TBA clusters spanning the right anterior and medial-temporal lobes. Medial temporal lobe (MTL) structures support memory functions and have been implicated in interference control^66,67^. Increased TBA in the most challenging trials likely indicates the involvement of the MTL in proactive interference control and successfully resolving the possibly detrimental effects of subliminal distractors^66^. Furthermore, the flanker effect following both atDCS and atDCS + MPH was associated with TBA in the right and left temporal pole (TP) respectively. When interference resolution is required, the right TP (i.e. a high-level visual cortical area) has been associated with object concept categorization based on their visual features^68,69^. Generally, the TP has been ascribed a role in receiving and integrating sensory modalities including visual inputs and participation in the ventral visual stream^70^. Ultimately, our results support the notion that in effortful situations the interaction of (mid-superior) frontal areas with MTL structures becomes more important^71^.

### Shared conflict effects

Overall, in high-conflict scenarios, both response conflicts were reflected in gain-induced ABA and TBA modulations in similar, yet critically distinct, neuroanatomical regions. Consistent with our hypothesis, subconsciously and consciously evoked conflicts (in the presence of the respective other conflict) were associated with activity modulations in the (pre)SMA in both frequency bands, likely reflecting gain control^29,72^. Mid-superior frontal cognitive control-related brain activity^29,37,54^), likely reflects goal shielding processes essential for stabilizing goal representations amidst distracting input^73,74^. Here, the presence of both conflicts likely potentiated the conflict effects given a certain threshold was surpassed^34^. When modulating gain control with atDCS, ABA and TBA were increased in conflicting, as compared to non-conflicting trials^37,54,62^, irrespective of catecholaminergic stimulation. This indicates enhanced gain control, where neuronal gain is adjusted to prioritize relevant information and effortfully suppress distractions. These gain control increases were reflected in negative clusters for both response conflicts, pharmacological groups, and frequency bands.

### Limitations

The observed results support the assumption that tDCS not solely affects the targeted cortical region, but also indirectly influences remote areas through interconnected cortical networks^75,76^. Given the evident cortical network-level impact of tDCS, this study further highlights the importance of interpreting tDCS effects within the context of broader neural interactions. A potential limitation of our study might have been that we did not assess whether participants could discern whether they were in the placebo or MPH group, nor which session involved active versus sham tDCS. It will further remain somewhat unclear whether the observed order effects are due to task learning, automatization, or both.

## Conclusions

In summary, we dissociated the modulatory effect of gain control stimulated with atDCS and atDCS + MPH on subliminally and consciously induced response conflicts. Combining catecholaminergic enhancement with atDCS resulted in greater gain-induced cognitive control and lower response conflicts for both subconscious and consciously induced conflicts compared to atDCS alone, especially when both conflict types were present. Following active atDCS (irrespective of MPH stimulation), both response conflicts were associated with larger conflict-induced ABA and TBA modulations (reflecting gain control) spanning mid-superior-frontal areas. Activity modulations in the SMA likely indicate the need to correct cued response tendencies by generating internal (correct) responses that overwrite stimulus-triggered movements. Gain control modulation (via atDCS and atDCS +MPH), induced similar neuronal gain task requirement-specific allocations of ABA and TBA. The overlapping modulatory pattern in the same cortical network subserving response conflict resolution substantiates the theoretical assumption that atDCS and MPH exert their effects through a shared underlying mechanism, specifically neuronal gain control^29,72^.

## Methods

### Participants

An a priori estimation of the required sample size using G*Power software^77,78^ assuming a within-inbetween interaction in a 4 × 8 ANOVA design (for details on the factors, please refer to the section on behavioral statistics) with α = 5%, 1-β = 95%, a correlation of 5, the assumption of non-sphericity and an effect size of 2% (i.e., ${\eta }_{p}^{2}=.02$, or *f* = 0.14285) yielded a required total sample size of *n* = 100. Of note, this sample size is comparable to another publication of ours investigating the combined effects of tDCS and (a higher dose of) MPH on response inhibition, where the smallest significant pharmacological effect explained around 4% of variance (i.e., twice as much as assumed for the a priori sample size estimation)^29^. *N* = 105 healthy adults (mean age 25.06 ± 0.288 SEM; 54 females) voluntarily participated in this study. All participants were 20-31 years old, had normal or corrected-to-normal vision, had no current/reported history of psychiatric, neurologic, or developmental disorders, and reported no CNS-affecting medication use. Participants provided written informed consent and received course credits or financial compensation. The study was approved by the ethics committee of TU Dresden (EK 420092015) and conducted following the Declaration of Helsinki and its later amendments. All ethical regulations relevant to human research participants were followed.

### Experimental design and methylphenidate administration

The study employed a mixed design with anodal tDCS (real vs. sham) and experimental conditions as within-subject factors, and pharmacological group (placebo vs. MPH) and order of stimulation (active tDCS on the first vs. second appointment) as between-subject factors. Pseudorandomization ensured equal numbers in each subgroup, defined by pharmacological group and order of stimulation. Group assignment was otherwise random and double-blind.

**Fig. 5:**
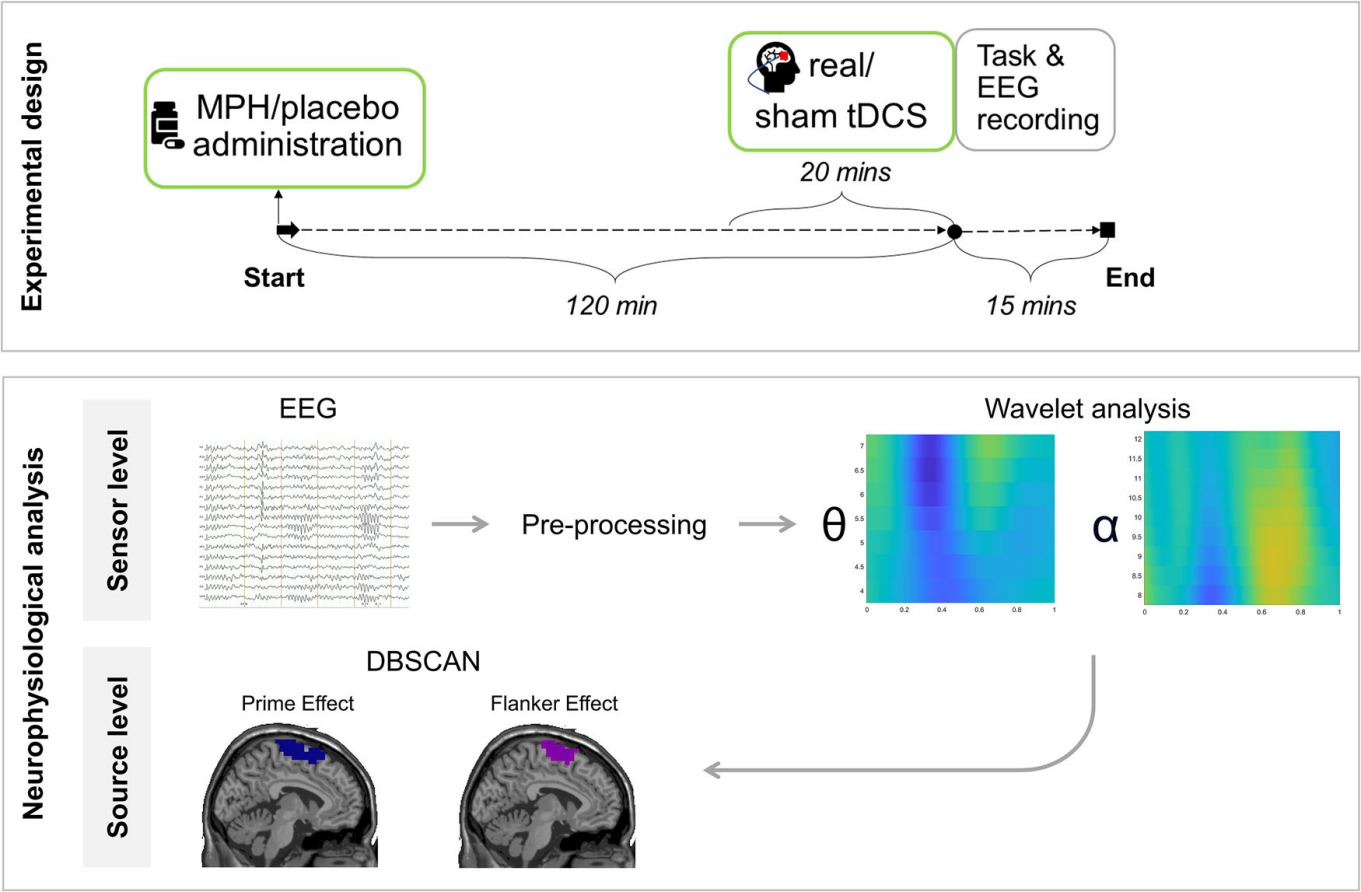
Methods overview. Illustrated is a visual summary of all methods and analyses applied in the current study. The top part depicts the timeline of an experimental session, showing the sequence and duration of key interventions. The horizontal axis marks time in minutes. Each session began with administering either a dose of MPH or a placebo, depending on the participants’ subgroup. Before starting the experimental task and EEG recording, participants received active/real tDCS on one appointment, and sham tDCS on the other (for details see methods section). Behavioral and neurophysiological data collection started 120 min post MPH/placebo administration and lasted approximately 15 min for the task reported in this study. Of note, this was followed by another task (duration ~20 min) that is not reported here. All details are provided in the methods section. The bottom part summarizes the analyses applied from the sensor to the source level. Abbreviations: tDCS (transcranial direct current stimulation), MPH (methylphenidate).

Participants attended two experimental sessions, spaced seven days apart. Depending on their randomly assigned pharmacological group, participants received either their respective MPH dose (0.50 mg per kg body weight) or a placebo (lactose) on both study appointments in a double-blind manner. Participants were instructed to abstain from caffeine and smoking for a minimum of two hours before testing. The experimental task on both appointments started roughly 120 min after drug administration, as this is when MPH plasma levels peak (MPH plasma levels peak around 1–3 h, with maximum drug concentration at about 2 h after oral administration^79,80^). A visual summary is provided in Fig. 5.

### tDCS stimulation

**Fig. 6:**
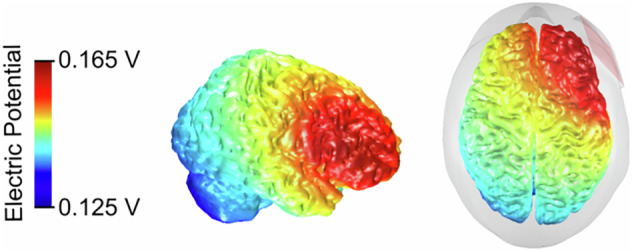
Illustration of a computational model of tDCS-induced electrical potential (EP) and anode placement. The EP induced by anodal tDCS with the described electrode configuration was computed using the “COMETS2” toolbox^83^. The anode (5 × 5 cm) was positioned above the rIFG, centered between electrodes FC4 and F8 (red patch in the right image). The reference electrode was positioned contralaterally between the neck and deltoid muscle. The input current was 2 mA, with colors denoting simulated electrical potential. Abbreviations: tDCS (transcranial direct current stimulation), rIFG (right inferior frontal gyrus), mA (milliampere).

Before the experiment and EEG recording, participants received 20 min of either anodal tDCS or sham stimulation targeting the right inferior frontal gyrus (rIFG), as shown in Fig. 5. Following previous studies^29,81,82^, the rIFG was identified as the midpoint between electrodes FC4 and F8, corresponding to the midpoint of the anode in our setup. The cathode (reference electrode) was positioned contralaterally between the neck and deltoid muscle. Anodal and sham stimulation were administered via two 5 × 5 cm² rubber electrodes (NeuroConn, Ilmenau, Germany) using a NeuroConn DC-Stimulator Plus device (NeuroConn, Ilmenaum Germany). Ten20 conductive paste (0.5 mm thick, customized casing^4^) ensured optimal conductivity and kept impedances below 5 kΩ. The current was ramped up and down over 15 sec to minimize possible discomfort, with active stimulation lasting 1200 s (20 min) at 2 mA. For the sham condition, a 2-mA current was applied for 15 s and then tapered off for 15 s to mimic the initial tingling of active tDCS. Both the tDCS protocols included identical waiting and preparation periods preceding the experiment. While the administration of MPH/placebo was double-blind, the experimenter could not be blinded to the tDCS manipulation, as they had to manually start the active or sham stimulation on the tDCS device. This detail was not disclosed to the participants to minimize potential biases. The electrical potential induced by the tDCS using the specified electrode configuration was calculated using the MATLAB toolbox “COMETS2”^83^ and is depicted in Fig. 6.

### Experimental task

**Fig. 7:**
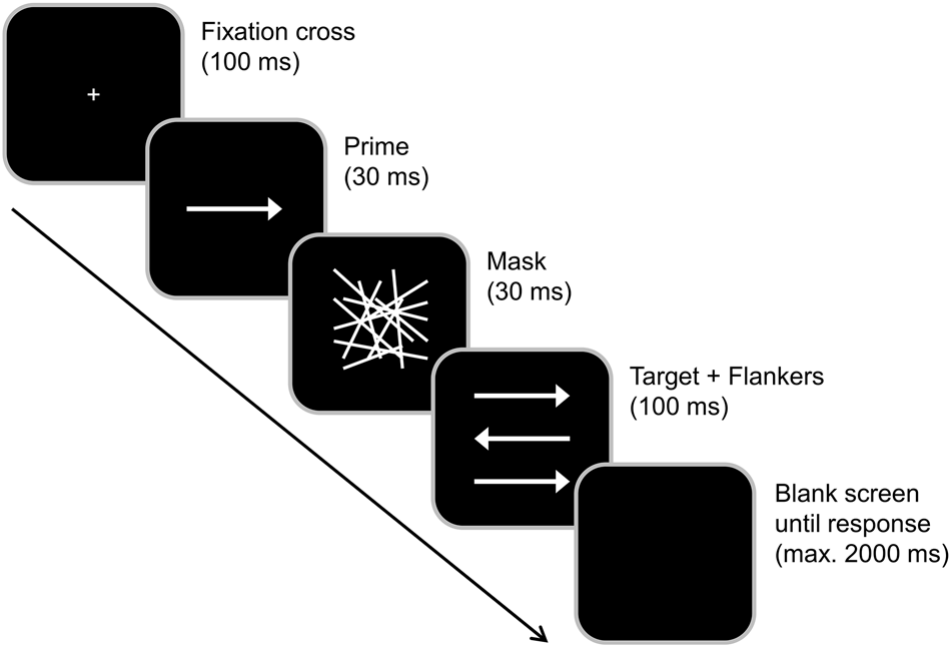
Boy Task. Illustrated is an exemplary trial configuration. Each trial began with a fixation cross displayed for 100 ms. This was followed by a prime (central arrow) for 30 ms and a mask (randomly allocated lines) for 30 ms. The target (central arrow) and flankers were presented for 100 ms, after which the screen turned black. Primes aligning with the target arrow were labeled compatible, while those in the opposite direction were incompatible. Similarly, flankers matching the target arrows’ direction were classified as congruent, and those pointing in opposite directions as incongruent.

The task design and structure are described and depicted in Fig. 7. The experimental task was adapted from Boy et al.^35^ and is identical to the paradigm utilized in previous studies^6,34,47^. This task allows for the examination of subconsciously and consciously evoked conflicts in response selection by combining the target stimulus with subliminal prime and flankers, respectively.

Participants were seated approximately 60 cm from a 24-inch CRT monitor, which displayed stimuli against a black background. Manual responses were collected using the “Ctrl” buttons on a standard QWERTZ keyboard. “Presentation” software (Version 18.3 by Neurobehavioral Systems, Inc.) managed stimulus presentation, response recording, and EEG synchronization. Each trial began with a central white fixation cross for 100 ms, followed by a subliminal prime (a white arrow pointing left or right) displayed centrally for 30 ms. Immediately after the prime, a mask stimulus (randomly distributed white lines) was shown for 30 ms. This resulted in a stimulus onset asynchrony (SOA) of 60 ms between the onsets of the subliminal prime and the target arrow. Subsequently, the target (a central white arrow pointing either left or right) and two flankers (white arrows positioned above and below the target arrow) were presented simultaneously for 100 ms. Participants were instructed to focus on the central target arrow and ignore the flankers. They were then asked to indicate the direction of the central target arrow by pressing the left “Ctrl” button with their left index finger for a left-pointing target arrow, or the right “Ctrl” button with their right index finger for a right-pointing target arrow. Each trial ended with the participant’s first response or 2000 ms after target onset. If no response was recorded within this time frame, the trial was marked as a “miss”. The response-stimulus interval (RSI) between the participant’s first response and the onset of the following trial varied randomly between 1000 and 1200 ms. Trials were categorized based on the direction of both the prime and target arrows. When the prime and target arrows pointed in the same direction, the trial was labeled as “compatible”. Conversely, if they pointed in opposite directions, the trial was labeled “incompatible”. Additionally, trials were classified as “congruent” when the flanker and target arrow pointed in the same direction, and as “incongruent” when they pointed in opposite directions. This classification resulted in four conditions: compatible-congruent, incompatible-congruent, compatible-incongruent, and incompatible-incongruent.

Each participant completed 384 trials, equally distributed across four blocks (96 trials per block). Within each block, all possible combinations of prime compatibility, flanker congruency, and target direction were randomized and presented at equal frequencies. At the first appointment, participants completed a practice run of 16 trials to familiarize themselves with the task immediately after MPH/placebo administration (i.e., before the potential onset of full MPH drug effects). Participants were instructed to respond as quickly and precisely as possible. After each block (96 trials), participants could take a self-timed break (i.e., to rest their eyes), and resume via button press. The experiment took on average approximately 15 min to complete.

### EEG recording and analysis

EEG signals were recorded from 60 Ag/AgCl electrodes in equidistant positions, as in previously published publications^54,84^. The EEG preprocessing was performed using the Automagic toolbox^85^ and the EEGLAB toolbox^86^ in Matlab 2020a (The MathWorks Corp.):

The participants’ EEG signals were recorded from 60 Ag/AgCl electrodes in equidistant positions with a “QuickAmp” amplifier (Brain Products GmbH, Gilching, Germany) and the “BrainVision Recorder” software (Version 2.2), as in previous publications from our research group^54,84^. For this, 60 Ag/AgCl electrodes in equidistant positions were used. The ground electrode was positioned at the coordinates θ = 58, φ = 78, and the reference at Fpz (θ = 90, φ = 90). EEG signals were initially recorded at a sampling rate of 500 Hz, while electrode impedances were kept below 10 kΩ. First, EEG data were down-sampled to 256 Hz, and flat channels were removed (i.e., channels that showed activity below 5 µV for more than 5 sec). The remaining channels were then re-referenced to an average reference. Subsequently, the PREP preprocessing pipeline^87^ was applied, which removes line noise (for data recorded in Europe: 50 Hz) using a multi-taper algorithm. After removing contaminations by noisy/bad channels (using high and minimum variance criterion), a robust common average reference was applied. EOG artifacts were removed using a subtraction method, i.e., EOG Regression^88^. Subsequently, the EEGLABs pop_eegfiltnew() pipeline was used to apply a high pass filter (cutoff frequency: 0.5 Hz) and low pass filter (cutoff frequency: 40 Hz); the filter order was estimated by default. Remaining artifactual source components in the data were detected by applying the Multiple Artifact Rejection Algorithm (MARA^89^), which automatizes independent component analyses (ICA). For the ICA, the data was temporarily high pass filtered with 1 Hz, but this option was not applied to the final pre-processed data. In the final step, removed/missing channels were interpolated using a spherical method.

After visually inspecting each dataset, the preprocessed data was imported into the FieldTrip toolbox^90^ for further analysis. The EEG data was segmented in a target-locked manner into the four trial conditions: compatible prime - congruent flanker, incompatible prime - congruent flanker, compatible prime - incongruent flanker, and incompatible prime - incongruent flanker. Only correct trials were considered for the subsequent analyses.

To examine alpha-band (8–12 Hz) and theta-band (4–7 Hz) activity during these conditions, time-frequency (TF) decomposition was performed using Morlet wavelets (with a width parameter of 5). The average power in the alpha and theta frequency bands was computed for each electrode and time point. Only significant and most relevant behavioral effects were further investigated at the neurophysiological level. For this approach, cluster-based permutation tests (CBPTs) were conducted to identify clusters of electrodes with significant differences between congruent and incongruent flanker trials in case of incompatible primes, as well as to identify significant differences between compatible and incompatible prime trials in case of incongruent flankers. The CBPTs were performed separately for each frequency band within 0-1000 ms following target arrow stimulus onset using 1000 Monte Carlo randomizations and a cluster-alpha level of *p* = .05 (two-tailed). Analyses were conducted separately for placebo and MPH groups for the active tDCS appointment.

### Source estimation and beamforming analysis

The neuroanatomical sources that reflect the prime effect (i.e., compatible minus incompatible) with incongruent flankers and the flanker effect (i.e., the congruent minus incongruent) with incompatible primes in the alpha and theta band were reconstructed following a previously established beamforming procedure^39^. First, a Dynamic Imaging of Coherent Sources (DICS) beamformer^91^ was used to identify neuroanatomical sources of substantial differences between conditions of interest in alpha and theta band activity. The source localization results were projected onto an equally spaced 0.5 cm grid created from the forward model template provided by the FieldTrip toolbox, based on the standard MNI (Montreal Neurological Institute) space. Power in the alpha and theta bands were extracted for the period following the presentation onset of the target stimulus. For both alpha and theta source power differences, corresponding contrasts were calculated and normalized on the total power of the two conditions as a ratio^55^:

$$	{Prime}\; {effect}\left({with}\; {incongruent}\; {flanker}\right){ratio} \\ 	 = \frac{{{Power}}_{{{compatible}}\; {{prime}}\; {{incongruent}}\; {{flanker}}}-{{Power}}_{{{incompatible}}\; {{prime}}\; {{incongruent}}\; {{flanker}}}}{{{Power}}_{{{compatible}}\; {{prime}}\; {{incongruent}}\; {{flanker}}}+{{Power}}_{{{incompatible}}\; {{prime}}\; {{incongruent}}\; {{flanker}}}}$$

$$	{Flanker}\; {effect}\left({with}\; {{incompatible}}\; {prime}\right){ratio} \\ 	 =\frac{{{Power}}_{{{congruent}}\; {{flanker}}\; {{incompatible}}\; {{prime}}}-{{Power}}_{{{incongruent}}\; {{flanker}}\; {{incompatible}}\; {{prime}}}}{{{Power}}_{{{congruent}}\; {{flanker}}\; {{incompatible}}\; {{prime}}}+{{Power}}_{{{incongruent}}\; {{flanker}}\; {{incompatible}}\; {{prime}}}}$$

Next, clusters of both frequency bands were identified by applying the density-based spatial clustering of applications with noise (DBSCAN^92^) algorithm as employed in MATLAB, comparable to previous studies^39,93^. The DICS beamforming results were restricted to negative ratios, indicating that alpha and theta power were higher in incompatible > compatible and incongruent > congruent trials. The negative top 2% of the power distribution in the prime and flanker effect ratio within labeled regions on the automated anatomical labeling atlas^94^ were submitted to the DBSCAN, restricting the analysis to the voxels with the largest negative differences. An epsilon of 1.5 the edge length of each voxel was used to detect neighboring voxels. The resulting clusters (minimum five voxels) were visually inspected.

### Statistics and reproducibility

We provide a power analysis justifying the recruited sample size (see section on participants for details). The behavioral data (i.e., response accuracy and reaction times) was analyzed using repeated measured ANOVAs using SPSS version 29.0.0.0 (IBM Corp., Armonk, N.Y., USA). As within-subject factors, the “tDCS” (active tDCS vs. sham), “prime compatibility” (compatible vs. incompatible), and “flanker congruency” (congruent vs. incongruent) were used. The between-subject factors were “pharmacological group” (placebo vs. MPH) and “order of stimulation” (active tDCS on the first appointment vs. active tDCS on the second appointment). Significant main or interaction effects were examined with post hoc ANOVAs and post-hoc t-tests. Given that post hoc tests were only conducted when a justifying significant interaction had been obtained in the ANOVA, post hoc tests were not corrected for multiple testing. Only correct trials, with response times (RTs) between min. 100 ms and max. 1000 ms after the target arrow onset, were included in the subsequent analyses. When a participant’s behavioral accuracy was below chance level (<50%) in two or more task conditions, or average response times were <100 ms/>1000 ms, that case was marked as an outlier, and no longer considered for all subsequent analyses of the behavioral and neurophysiological data. Descriptive data are given as mean and standard error of the mean (SEM). We used cluster-based permutation tests for the EEG data analysis (see section on EEG recording and analysis). The tDCS approach was based on prior research and a modeling of the tDCS current. EEG-beamforming analysis was only performed for contrasts for the cluster-based permutation testing revealed robust effects and according to previously published protocols.

### Reporting summary

Further information on research design is available in the Nature Portfolio Reporting Summary linked to this article.

## Supplementary information

Supplementary Material

Reporting Summary

## Data Availability

All data from this study are publicly accessible at the following link: https://osf.io/wexgu/^95^. For any additional inquiries, please contact Anna Helin Koyun (AnnaHelin.Koyun@ukdd.de).

## Code availability

The custom scripts used for EEG data processing are available for download at: https://osf.io/wexgu/.
