# Supplementary material for: Neurophysiological insights into catecholamine-dependent tDCS modulation of cognitive control
Source: Commun Biol. 2025 Mar 6;8:375. doi: 10.1038/s42003-025-07805-6 (PMC11885824; doi:10.1038/s42003-025-07805-6)
Supplement: Supplementary file 1 — Supplementary Material [file 42003_2025_7805_MOESM1_ESM.pdf]

Supplementary Material

Publication Title: ***Neurophysiological Insights into Catecholamine-Dependent tDCS Modulation of Cognitive Control***

Authors: *Anna Helin Koyun, Paul Wendiggensen, Veit Rößner, Christian Beste, Ann-Kathrin Stock*

Description of the experimental task

Participants were seated approximately 60 cm from a 24-inch CRT monitor, which displayed stimuli against a black background. Manual responses were collected using the "Ctrl" buttons on a standard QWERTZ keyboard. "Presentation" software (Version 18.3 by Neurobehavioral Systems, Inc.) managed stimulus presentation, response recording, and EEG synchronization. Each trial began with a central white fixation cross for 100 ms, followed by a subliminal prime (a white arrow pointing left or right) displayed centrally for 30 ms. Immediately after the prime, a mask stimulus (randomly distributed white lines) was shown for 30 ms. This resulted in a stimulus onset asynchrony (SOA) of 60 ms between the onsets of the subliminal prime and the target arrow. Subsequently, the target (a central white arrow pointing either left or right) and two flankers (white arrows positioned above and below the target arrow) were presented simultaneously for 100 ms. Participants were instructed to focus on the central target arrow and ignore the flankers. They were then asked to indicate the direction of the central target arrow by pressing the left "Ctrl" button with their left index finger for a left-pointing target arrow, or the right "Ctrl" button with their right index finger for a right-pointing target arrow. Each trial ended with the participant's first response or 2000 ms after target onset. If no response was recorded within this time frame, the trial was marked as a “miss”. The response-stimulus interval (RSI) between the participant's first response and the onset of the following trial varied randomly between 1000 and 1200 ms. Trials were categorized based on the direction of both the prime and target arrows. When the prime and target arrows pointed in the same direction, the trial was labeled as “compatible”. Conversely, if they pointed in opposite directions, the trial was labeled “incompatible”. Additionally, trials were classified as “congruent” when the flanker and target arrow pointed in the same direction, and as “incongruent” when they pointed in opposite directions. This classification resulted in four conditions: compatible-congruent, incompatible-congruent, compatible-incongruent, and incompatible-incongruent.

Each participant completed 384 trials, equally distributed across four blocks (96 trials per block). Within each block, all possible combinations of prime compatibility, flanker congruency, and target direction were randomized and presented at equal frequencies. At the first appointment, participants completed a practice run of 16 trials to familiarize themselves with the task immediately after MPH/placebo administration (i.e., before the potential onset of full MPH drug effects). Participants were instructed to respond as quickly and precisely as possible. After each block (96 trials), participants could take a self-timed break (i.e., to rest their eyes), and resume via button press. The experiment took on average approximately 15 minutes to complete.

EEG recording and preprocessing

The participants’ EEG signals were recorded from 60 Ag/AgCl electrodes in equidistant positions with a “QuickAmp” amplifier (Brain Products GmbH, Gilching, Germany) and the “BrainVision Recorder” software (Version 2.2), as in previous publications (Koyun et al., 2023, 2024 & other publications from our research group). For this, 60 Ag/AgCl electrodes in equidistant positions were used. The ground electrode was positioned at the coordinates $\theta = 58$, $\varphi = 78$, and the reference at Fpz ($\theta = 90$, $\varphi = 90$).

EEG signals were initially recorded at a sampling rate of 500 Hz, while electrode impedances were kept below 10 kΩ. First, EEG data were down-sampled to 256 Hz, and flat channels were removed (i.e., channels that showed activity below 5 µV for more than 5 sec). The remaining channels were then re-referenced to an average reference. Subsequently, the PREP preprocessing pipeline (Bigdely-Shamlo et al., 2015) was applied, which removes line noise (for data recorded in Europe: 50 Hz) using a multi-taper algorithm. After removing contaminations by noisy/bad channels (using high and minimum variance criterion), a robust common average reference was applied. EOG artifacts were removed using a subtraction method, i.e., EOG Regression (Parra et al., 2005). Subsequently, the EEGLABs pop_eegfiltnew() pipeline was used to apply a high pass filter (cutoff frequency: 0.5 Hz) and low pass filter (cutoff frequency: 40 Hz); the filter order was estimated by default. Remaining artifactual source components in the data were detected by applying the Multiple Artifact Rejection Algorithm (MARA; Winkler et al., 2011), which automatizes independent component analyses (ICA). For the ICA, the data was temporarily high pass filtered with 1 Hz, but this option was not applied to the final pre-processed data. In the final step, removed/missing channels were interpolated using a spherical method.

Source estimation and beamforming analysis

First, dynamic imaging of coherent sources (DICS) beamforming (Gross et al., 2001) was applied to identify neuroanatomical sources of substantial differences between conditions of interest in the frequency domain. The source localization results were projected onto an equally spaced 0.5 cm grid created from the forward model template provided by the FieldTrip toolbox, based on the standard MNI (Montreal Neurological Institute) space. Power in the alpha and theta bands were extracted for the period following the presentation onset of the target stimulus. For both alpha and theta source power differences, corresponding contrasts were calculated and normalized on the total power of the two conditions as a ratio (Mückschel et al., 2016):

$$Prime\ effect\ (with\ incongruent\ flanker)\ ratio = \frac{Power_{compatible\ prime\ incongruent\ flanker} - Power_{incompatible\ prime\ incongruent\ flanker}}{Power_{compatible\ prime\ incongruent\ flanker} + Power_{incompatible\ prime\ incongruent\ flanker}}$$

$$Flanker\ effect\ (with\ incompatible\ prime)\ ratio = \frac{Power_{congruent\ flanker\ incompatible\ prime} - Power_{incongruent\ flanker\ incompatible\ prime}}{Power_{congruent\ flanker\ incompatible\ prime} + Power_{incongruent\ flanker\ incompatible\ prime}}$$

Next, clusters of both frequency bands were identified by applying the density-based spatial clustering of applications with noise (DBSCAN; Ester et al., 1996) algorithm as employed in MATLAB, comparable to previous studies (Adelhöfer & Beste, 2020; Wendiggensen et al., 2022). The DICS beamforming results were restricted to negative ratios, indicating that alpha and theta power were higher in incompatible > compatible and incongruent > congruent trials. The negative top 2% of the power distribution in the prime and flanker effect ratio within labeled regions on the automated anatomical labeling atlas (Tzourio-Mazoyer et al., 2002) were submitted to the DBSCAN, restricting the analysis to the voxels with the largest negative differences. An epsilon of 1.5 the edge length of each voxel was used to detect neighboring voxels.

Additional details on exclusion decisions and the composition of the final sample

After initial data inspection, n = 12 participants were excluded from all subsequent analyses (behavioral and EEG data), due to the following reasons: n = 1 participant dropped out after the first appointment, n = 2 participants' response accuracy was below chance level (< 50%), n = 1 participant was marked as

a reaction time outlier, n = 2 participants were excluded due to incomplete data, n = 3 participants were excluded due to technical problems with the tDCS device, and n = 3 participants were excluded due to insufficient/noisy EEG signal quality.

Additional details on behavioral data: Learning/practice effects

After having found order-dependent effects in the behavioral analyses reported in the main manuscript, we ran add-on analyses to determine the overall size of practice / learning effects. For this purpose, we averaged all task conditions and compared the performance data collected on the first study appointment to that collected on the second study appointment using paired t-tests. Doing so, we found significantly better performance in the second, as compared to the first appointment for both response accuracy [$t_{(92)}$ = 2.541; p = .006 (one-sided p); first appointment = 95.5 % ± 0.4; second appointment = 96.4 % ± 0.4] and response times [$t_{(92)}$ = 4.194; $p < .001$ (one-sided p); first appointment = 408 ms ± 4; second appointment = 397 ms ± 4].

Additional neurophysiological results for participants who received sham tDCS on the first appointment

In the 0−1000 ms interval relative to target onset, separate cluster-based permutation tests (CBPTs) were conducted for alpha and theta band activity (ABA and TBA respectively), and MPH and placebo groups. The results revealed significant negative differences in both ABA and TBA between compatible and incompatible primes trials when the flankers were incongruent and between congruent and incongruent flanker trials when the prime was incompatible. Negative power differences indicate that alpha and theta band power were higher in incompatible prime trials compared to compatible ones, and in incongruent flanker trials compared to congruent ones, potentially highlighting greater difficulty or interference in these conditions. The significant electrode locations of the cluster-based permutation tests (CBPTs) are visualized in **Supplementary Figures 1 a and b**, accompanied by the time-frequency representation (TFRs) of the power differences in the aforementioned contrasts. For the Flanker effect in incompatible prime trials in the alpha band, no significant electrodes were revealed by the CBPTs for the placebo group, thus not visualized.

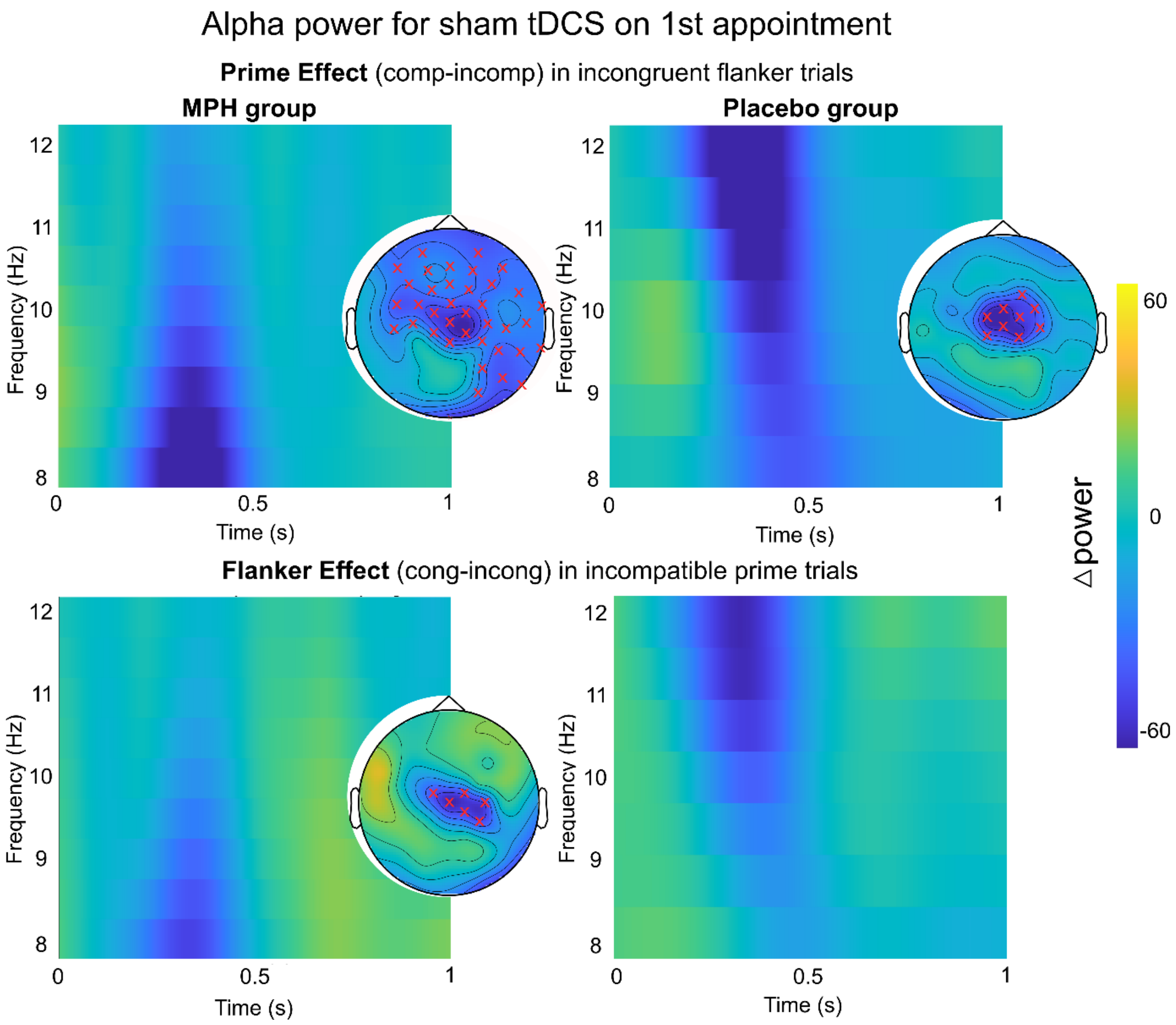

Supplementary Figure 1 a. Results of the time-frequency (TF) analyses (TF plots) and the cluster-based permutation testing (topographic plots) for the alpha band.

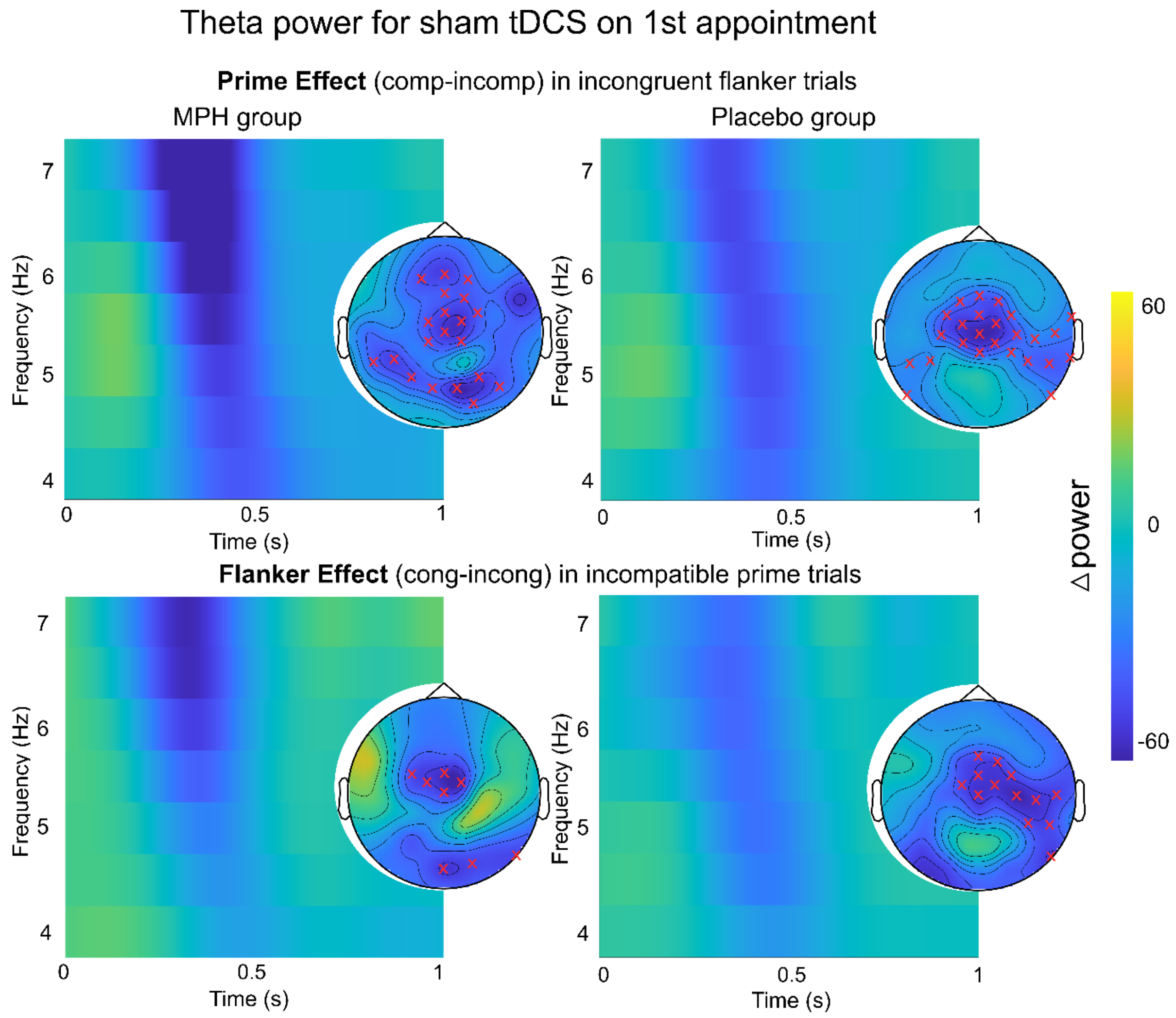

Supplementary Figure 1 b. Results of the time-frequency (TF) analyses (TF plots) and the cluster-based permutation testing (topographic plots) for the theta band.

On the source level, the DBSCAN algorithm on the Prime and Flanker effect ratio (see "*Source estimation and beamforming analysis*") identified several negative clusters in both alpha and theta bands for both MPH and placebo groups. On the sensor level, for the Flanker effect in incompatible prime trials in the alpha band, no significant electrodes were revealed by the CBPTs for the placebo group, thus no sources could be located.

Negative clusters (i.e., negative power ratios) indicate that ABA and TBA were higher in incompatible prime trials compared to compatible ones, and in incongruent flanker trials compared to congruent ones. An overview of all resulting clusters is provided in **Supplementary Figures 2 a and b**.

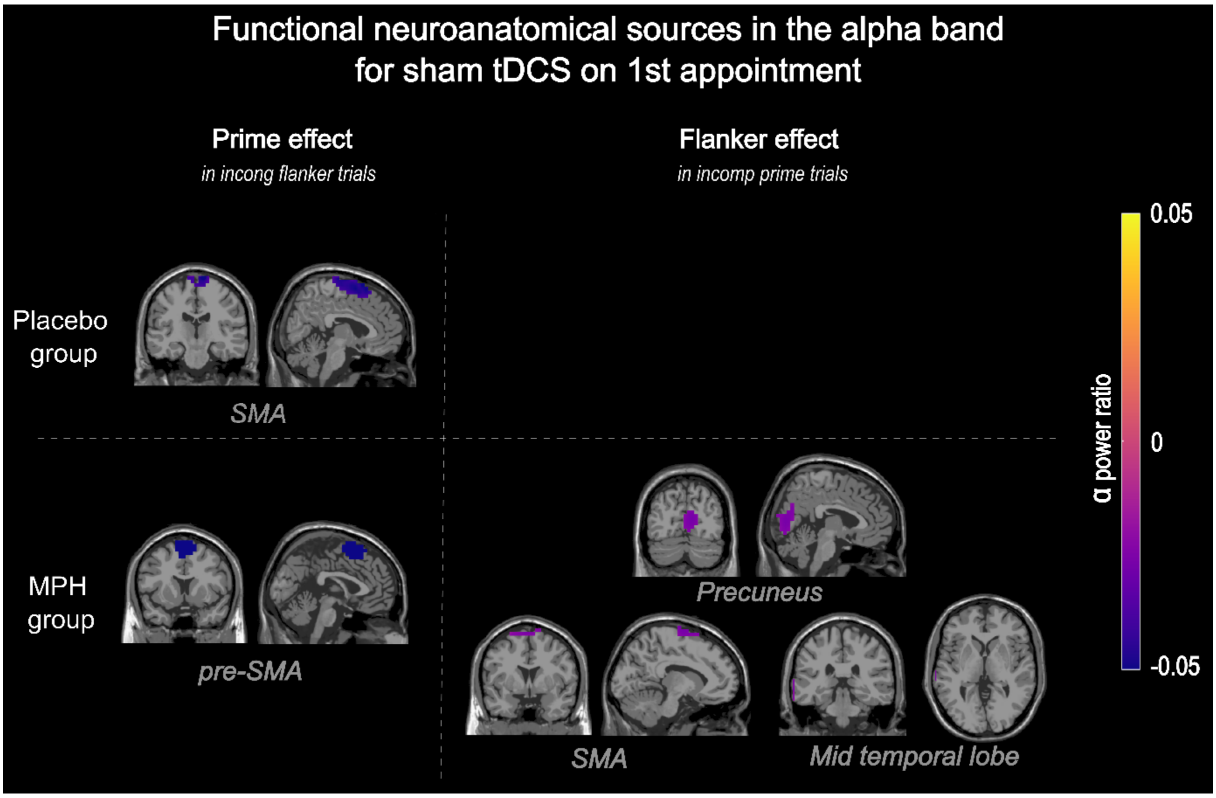

Supplementary Figure 2 a. Source localization results for the alpha band.

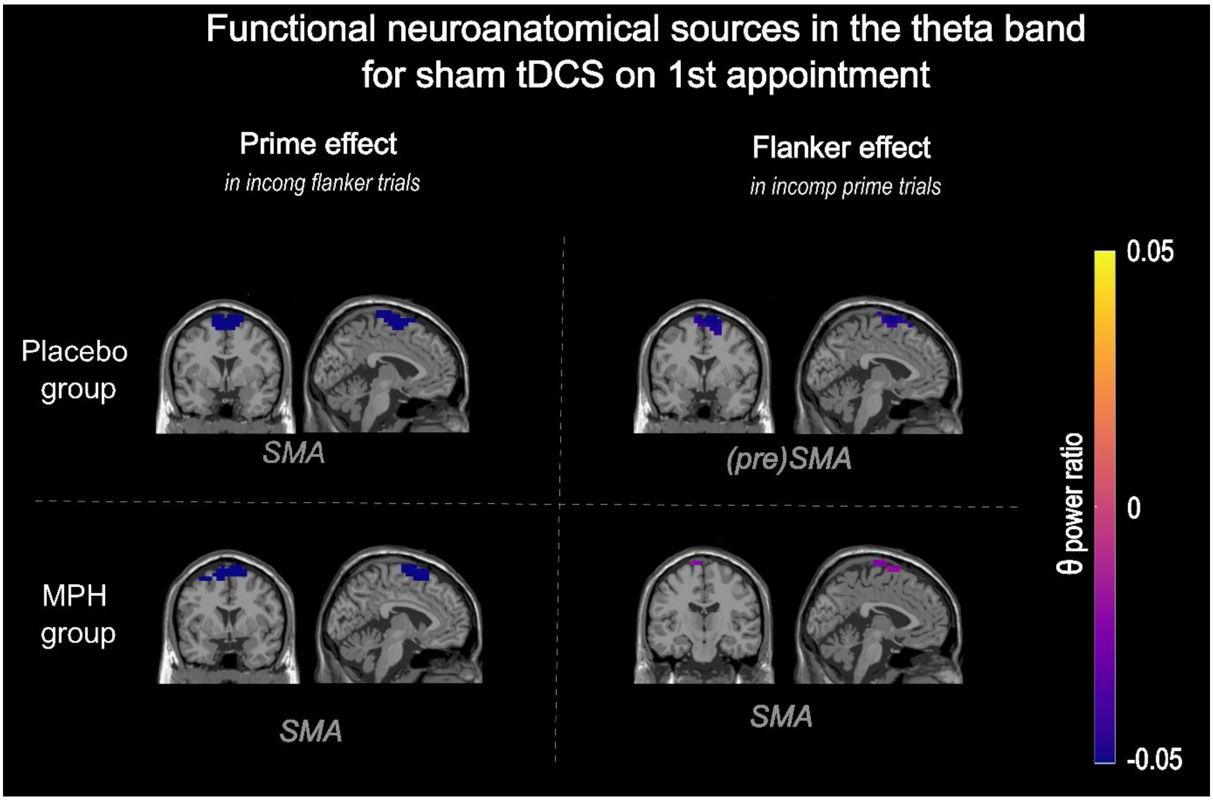

Supplementary Figure 2 b. Source localization results for the theta band.
